# Early Nutrition Impacts on Growth, Skeletal Anomalies and Organ Ontogeny in Larval Atlantic Cod (*Gadus morhua*)

**DOI:** 10.3390/ani15202985

**Published:** 2025-10-15

**Authors:** Joana Pedro, João Henriques, Maria Bergvik, Konstantinos Tzakris, Michael Viegas, Katerina Loufi, Jorge M. O. Fernandes, Benjamín Costas, Nils Tokle, Luís E. C. Conceição

**Affiliations:** 1 CIIMAR-Interdisciplinary Centre of Marine and Environmental Research, 4450-208 Matosinhos, Portugal; joana.pedro@sintef.no (J.P.); bcostas@ciimar.up.pt (B.C.); 2 SINTEF Ocean, 7010 Trondheim, Norway; maria.bergvik@sintef.no; 3 SPAROS, Lda., 8700-221 Olhão, Portugal; joaohenriques@sparos.pt; 4 Ode AS, 7105 Stadsbygd, Norway; 5 Planktonic AS, 7018 Trondheim, Norway; kostas.tzakris@planktonic.no (K.T.); nils.tokle@planktonic.no (N.T.); 6 S2AQUA—Collaborative Laboratory, Association for a Sustainable and Smart Aquaculture, 8700-194 Olhão, Portugal; michael.viegas@s2aquacolab.pt; 7 Department of Biology, University of Patras, Rio Achaias, 265 04 Patras, Greece; aloufi@upatras.gr; 8 Faculty of Biosciences and Aquaculture, Nord University, 8049 Bodø, Norway; jorge.m.fernandes@nord.no; 9 Department of Renewable Resources, Institute of Marine Sciences (ICM-CSIC), 08005 Barcelona, Spain; 10 Instituto de Ciências Biomédicas Abel Salazar (ICBAS-UP), Universidade do Porto, 4050-313 Porto, Portugal

**Keywords:** live feeds, barnacle nauplii, microdiets, hatchery feeding protocols

## Abstract

**Simple Summary:**

Fish farming of Atlantic cod is important for preventing the decline of wild stocks, but ensuring young fish are healthy and grow well remains a challenge, particularly concerning early diet and preventing physical defects like skeletal anomalies. This study investigated a new early feeding strategy for Atlantic cod larvae, aiming to see if specialized live feeds (barnacle nauplii and plankton eggs) combined with two experimental dry feeds could improve overall fish quality, focusing on growth, survival, organ development, and skeletal anomalies. A control group (COM) and two experimental groups (D1 and D2) were used, using different live feeds and dry feeds with different vegetable versus marine fat composition. Although the control group showed slightly better final growth, the D1 feeding protocol resulted in dramatically healthier fish, reducing the occurrence of skeletal anomalies from 91 percent (COM) down to 52 percent and significantly reducing severe anomalies like scoliosis. This specialized diet also sped up the maturation and development of internal organs, such as the digestive tract and liver, compared to the other groups. These results highlight that optimizing early nutrition is crucial for producing high-quality cod juveniles, offering valuable insights for the sustainable growth of the aquaculture industry.

**Abstract:**

Early life nutrition is a critical factor influencing subsequent performance and quality, including skeletal development, in farmed Atlantic cod (*Gadus morhua*). This study investigated the effects of a novel start-feed protocol utilizing barnacle nauplii and plankton eggs and two experimental microdiets on larval survival, growth, skeletal anomalies, and organ ontogeny. Atlantic cod larvae were reared using three feeding protocols (COM, D1, and D2): COM used enriched rotifers and a commercial microdiet, while D1 and D2 protocols incorporated blue mussel eggs (Cryo-µ) and barnacle nauplii (Cryo-S, Cryo-L), followed by inert microdiets that differed in their phospholipid (PL) source (D1 richer in vegetable PL; D2 richer in marine PL). Larvae were sampled up to 66 days post hatching (dph) for morphometric, skeletal anomaly, and histological analyses. Survival averaged 21.3% and was unaffected by the diets. The control group had slightly higher standard length and dry weight at 66 dph compared to the experimental groups. However, larvae fed the D1 protocol exhibited a significantly lower overall prevalence of skeletal anomalies (52%) compared to the control group (91%). Moreover, D1 showed a lower occurrence of severe anomalies and a significantly reduced prevalence of scoliosis compared to both D2 and COM groups. Histology showed that group D1 achieved an overall accelerated organ ontogeny, with greater villi length and goblet cell abundance in the anterior intestine at 66 dph. In conclusion, the novel D1 feeding protocol, incorporating barnacle nauplii and a microdiet richer in vegetable phospholipids, enhanced larval quality by effectively reducing skeletal anomalies and accelerating internal organ development.

## 1. Introduction

Atlantic cod (*Gadus morhua*) is a very relevant species in the North Atlantic Ocean fisheries and has contributed to the prosperity of several nations in this region [1]. The decline in cod stocks in the late 1970s and the 1990s, particularly in Norway, and the restrictions on fisheries [2] have spurred interest in cod farming [3]. Potential improvements exist within the environment to which farmed Atlantic cod is exposed, especially during early life stages. Nutrition during this stage has been shown to influence the later life performance in various organisms, including teleost fish such as Atlantic cod [4,5,6]. 

Live feed has been widely used in fish larvae nutrition, valued for its ease of detection due to movement and high digestibility, thus being more available and able to stimulate larval feeding responses [7]. Although enriched rotifers and *Artemia* spp. have been the main live prey used for fish larvae rearing [4,6,8], the inclusion of natural zooplankton, mainly copepods, in larvae live feed protocols has resulted in better growth, survival, performance, and quality, in particular for Atlantic cod larvae [4,9,10,11,12]. Several studies have investigated the effects of the inclusion of natural zooplankton in Atlantic cod diets, which resulted in higher growth rates and viability [13,14,15], resulting in long-term effects [4,10]. It has been suggested that higher levels of taurine [14,16,17], iodine [13,16], and selenium [13,16], as well as the polar lipid fraction and fatty acid composition [6] provided by natural zooplankton, may be involved in the accelerated growth in fish larvae with optimal nutritional conditions. Besides copepods (Copepoda, Arthropoda), barnacles (Cirripedia, Crustacea) are also a potential live feed for marine larvae, having been tested with success in feeding protocols for ballan wrasse (*Labrus bergylta*) [18,19] and other fish species (unpublished results). Cirripedia nauplii are morphologically and nutritionally similar to the same life stage from copepods [18,20,21,22], and in addition, it is possible to cryo-preserve, thaw, and revitalize this plankton organism, allowing their use as moving live prey. Nutrient analyses of large barnacle nauplii (Cryo-L; *Semibalanus balanoides* n1) and small barnacle nauplii (Cryo-S; *Balanus crenatus* n1) have shown significantly higher levels of n-3 eicosapentaenoic acid (EPA, 20:5n-3), iodine, and proline and lower levels of arachidonic acid (ARA, 20:4n-6), compared to rotifers and *Artemia* spp. and docosahexaenoic acid (DHA, 22:6n-3) levels similar to *Artemia* spp. and rotifers [18,19]. When comparing the fatty acid profile of barnacles, copepods, *Artemia* spp. and rotifers, levels of ARA are similar between barnacles and copepods, while EPA is the highest in barnacles, with the overall profile being similar to copepods in later nauplii stages [18,22]. Moreover, both small and large barnacle nauplii have higher contents of taurine and vanadium (V), manganese (Mn), cobalt (Co), zinc (Zn), arsenic (As), selenium (Se), and calcium (Ca) when compared to rotifers [19]. 

Larvae can be weaned with an inert formulated diet after a few weeks, shortening the need for live feeds and providing a stable and better nutritionally balanced feed composition [11,23]. However, it is extremely important to develop adequate inert diets, bearing in mind a species’ nutritional needs, since the diet composition, digestibility, and absorption have effects on appetite, growth, digestive tract maturation, and skeletal development of marine larvae [8,23].

Besides the need to meet nutritional requirements, other characteristics influence microparticulate diets’ applicability. Small particle sizes may lead to high losses by leaching water-soluble nutrients, compromising the nutritional value of feed and water quality [11,24,25]. The successful use of microparticulate diets in combination with live feed potentiates growth and survival while reducing the weaning period and production costs [24]. The use of an extended co-feeding period with live feed has been proven to increase acceptance of microdiets [8,26], and early introduction of microdiets in larval feeding protocols can promote growth potential in the mid-term [23]. Still, excessive live feed replacement may also lead to losses in growth potential [27]. 

Despite much progress on larvae nutritional requirements [8,11,28,29,30], there are still many limitations regarding the knowledge on the larval optimal levels of nutrients, including for Atlantic cod larvae. Nevertheless, formulated feeds should ensure a balanced amino acid profile [31] and include hydrolyzed proteins [7] to ensure higher digestibility, prevent skeletal anomalies, and improve survival [32]. Additionally, appropriate levels of essential fatty acids (EFAs), which cannot be synthesized by the organism and must be obtained through the diet, such as DHA and EPA, are known to be essential to guarantee normal development, appropriate growth, and survival [8,24,26,32]. Furthermore, it has been emphasized that at least some of these EFAs should be present in the form of dietary phospholipids (PLs), since this is also what is described for natural zooplankton [3,8,28,29,30,33], reducing the need for enrichment and therefore improving larval quality, including a normal inflation of the swim bladder [12]. PLs have cell structural functions and are involved in dietary lipid utilization, thus being important for growth, gut maturation, and skeletal development [8].

Nutritional requirements have significant effects on organ ontogeny and skeletal development [8,33]. In fish with indirect development, such as Atlantic cod, larvae go through a remodeling process from larval to juvenile characteristics, referred to as metamorphosis [33]. The success of these adaptations is greatly influenced by nutrition, which impacts digestive ontogeny and, consequently, the possible occurrence of skeletal anomalies. Inappropriate levels of EFAs, PLs, vitamins, and minerals influence larval development and quality, including skeletal development and the occurrence of skeletal anomalies [8,29,34,35,36]. These result from the interaction of various factors and have detrimental effects on welfare, growth, survival, and, ultimately, product quality [8,10,37,38,39]. 

Therefore, the current study aimed to understand the potential of a novel start-feed protocol with barnacle nauplii and plankton eggs, along with two experimental microdiets, for survival, growth performance, skeletal anomalies, and organ ontogeny in Atlantic cod larvae.

## 2. Materials and Methods

### 2.1. Ethics Statement

All fish handling and sampling protocols comply with guidelines under the Norwegian Animal Welfare Act of 20 December 1974, No. 73, Sections 20–22, amended 19 June 2009, and the Guidelines of the European Union Council [40].

### 2.2. Cod Larvae Husbandry

Atlantic cod eggs were collected from a natural spawning broodstock population kept at the National Cod Breeding Center in Tromsø (Norway), transferred to Ode hatchery facilities (Stadsbygd, Norway), and placed into incubators with a capacity of 600 L for 10 days at 6.0 ± 0.5 °C. At 2 days post hatching (dph), 40,000 larvae were transferred to each of nine 400 L start-feeding tanks. The water parameters were monitored daily, maintaining oxygen saturation above 90%, salinity at 35 psu, and temperature at 8.8 ± 0.2 °C until 30 dph, followed by 10.8 ± 0.2 °C until 66 dph. Larvae were reared with a 24L:0D photoperiod with algae paste (*Nanochloropsis gaditana*, AlgaSpring, Almere, The Netherlands) additions from 2 to 47 dph.

### 2.3. Experimental Design and Diets

The experiment was run in triplicate with one control group and two experimental feeding protocols, fed according to the timeline in Table 1. The control diet (COM) consisted of rotifers enriched with Larviva Multigain (Biomar, Aarhus, Denmark), from 3 to 28 dph, and Cryo-L, from 20 to 45 dph. A commercial inert diet (Gemma Micro, Skretting, Stavanger, Norway) was introduced at 27 dph and co-fed with Cryo-L until 45 dph. From 46 dph onwards, larvae were fed solely with the referred formulated diet. The two experimental groups (D1 and D2) were also fed with enriched rotifers from 3 to 24 dph, blue mussel eggs (Cryo-µ; *Mytilus edulis* eggs) from 3 to 9 dph, Cryo-S from 10 to 25 dph, and Cryo-L from 20 to 45 dph. Two experimental formulated feeds (D1 and D2) were introduced at 27 dph, co-fed with Cryo-L until 45 dph, and, after this, maintained on formulated microdiets for the remainder of the trial (66 dph).

Small and large barnacle nauplii (Cryo-S, 200 µm, and Cryo-L, 320 µm) and blue mussel eggs (Cryo-µ, 60–70 µm) were provided by Planktonic AS (Trondheim, Norway) as CryoPlankton delivered in cryogenic dewars and ready to be fed after a preparation process of thawing, rinsing, and revitalization. The experimental inert feeds D1 and D2 were provided by Sparos Lda (Olhão, Portugal). 

The proximal compositions of the three inert diets applied during the experiment are given in Table 2. The two experimental diets differed only in the proportion of phospholipids (PL) from marine or vegetable origin. Diet D1 was richer in vegetable PL, while D2 was richer in marine PL. The main ingredients used in the inert diets D1 and D2 were squid meal, krill meal, fish hydrolysate, lecithin, wheat gluten, and krill oil. In turn, the COM inert diet had fish meal, hydrolyzed aquatic invertebrates, lecithin, wheat gluten, algae, and fish oil as main ingredients.

### 2.4. Sampling Procedure

Pooled samples of 30 whole larvae per tank were sampled randomly from each tank at 3, 30, 50, and 66 dph to determine standard length (SL) and dry weight (DW), relative growth rate (RGR), and feed conversion ratios (FCR). Sampled larvae were euthanized with an overdose of tricaine mesylate (MSD, Rahway, NJ, USA) dissolved in seawater. Larvae standard lengths were measured using a stereoscope (Leica, Wetzlar, Germany), immediately washed with distilled water, placed in aluminum foil, and stored at −20 °C until further analysis. Dry weight was determined by drying whole larvae samples to a constant weight at 104 °C for 24 h [41]. For histological analysis, pools of 6 larvae per tank were taken at 15, 30, and 66 dph, fixed, and stored in formaldehyde 4% until processed. In addition, at 66 dph, 25 larvae from each tank were collected for skeleton anomaly assessment. Larvae were washed two times in NaCl 0.9%, fixed in formaldehyde at 4% overnight at 4 °C, rinsed in distilled water, washed in NaCl 0.9%, and preserved in 75% ethanol for later processing.

### 2.5. Histological Procedures

Skeletal anomalies at 66 dph were identified using a modified double-staining procedure according to a previously described protocol [42]. Cartilaginous structures were stained with Alcian Blue 8GX (Sigma-Aldrich, Louis, MI, USA), followed by the staining of calcified structures with Alizarin Red S (Sigma-Aldrich, USA). This staining was performed in combination with KOH and peroxide treatment to increase the transparency of soft tissues and allow clear observation. The stained specimens were subsequently preserved in glycerol and photographed in a stereomicroscope equipped with a digital camera.

The nomenclature and the characterization of the different types of anomalies were performed according to Sæle et al. [38], Gavaia et al. [42], and Deschamps et al. [43] (Table 3).

For histological analysis, samples were placed in distilled water for one hour to rinse the formalin residuals and then dehydrated through a series of increasing concentrations of ethanol 70%, 80%, and 100% for one hour each. After dehydration, fish were embedded in a solution of methacrylate resin (Technovit 7100^®^, Heraeus Kulzer, Hanau, Germany) and ethanol 100% (1:1) for one hour, and in methacrylate resin for 24 h. The final step was the polymerization of the samples by embedding them in methacrylate resin and hardener for 24 h. The polymerized blocks were cut into 5 μm thick sections on a microtome (Leica SM2000R, Wetzlar, Germany), stained with methylene blue (Sigma, Darmstadt, Germany)/Azure II (Sigma, Germany)/Basic Fuchsin (Polysciences, Warrington, PA, USA) [44], and examined with a light microscope (Zeiss AX10 Image A2, Oberkochen, Germany) with ×100 and ×200 magnification. The image-analysis software Image J 1.54 (NIH, Bethesda, MD, USA) was utilized to measure all the indices in the liver, anterior intestine, the eye, and the gills, by using a modified version of the [45] technique. More precisely, six sections were represented by six distinct microphotographs. For the calculation of any type of cell, a line was manually drawn, and its length was calculated in pixels to determine cell abundance. The pixels were converted into micrometers and then the number of cells was calculated per 100 μm of tissue length. Regarding the calculation of the area covered by lipid vacuoles (ACLV) (%) in the posterior intestine and liver, six microphotographs from six different areas were obtained, and the color gradients (brightness and saturation) were adjusted to make lipid-vacuolized areas appear white. In the liver, any other tissues that could be confused by the software (Image J) as lipid vacuoles (e.g., glycogen) were manually excluded from the analysis according to [46]. Histological evaluation was carried out by utilizing a Multiparametric Semi-Quantitative Scoring System (MSSS), adjusted from the protocol in [47], in order to assess morphological indices, such as the length of the villi and the abundance of goblet cells in the anterior intestine, the ACLV in the posterior intestine and the liver, as well as pathological indices such as fusion, clubbing, and edema in the gills. This MSSS consisted of a 1–5 score, corresponding to absent, scarce, moderate, abundant, or highly abundant, according to the analyzed structure.

### 2.6. Statistical Analysis

Data were first checked for normality and homogeneity of variance using the Shapiro–Wilk W-test and Levene’s test, respectively. Outliers were removed from the analysis according to results from Grubbs’ test. When necessary, data were transformed to meet the assumptions of normality and homogeneity of variance. Significant differences between larval stages were determined using one-way ANOVA followed by Tukey’s multiple comparison test. When normality and homogeneity of the data were not achieved, a non-parametric Kruskal–Wallis test was applied. The level of significance for all statistical tests was *p* < 0.05. All statistics were performed with R-statistical software (Version 4.2.2) with specific packages including outliers, tidyverse (dplyr, tidyr, ggplot2), car, multcomp, psych, base, and MASS.

The relative growth rate (RGR, % weight day^−1^) was calculated as RGR = (e^g^ − 1) × 100, where e = exponential and g = (lnWf − lnWi) × t^–1^. Wf and Wi correspond to the final and initial weights, respectively. The feed conversion ratio (FCR) was calculated as FCR *=* (Fi/Wg), where Fi corresponds to feed intake (g) and Wg to the mean weight gain (g).

## 3. Results

### 3.1. Growth and Survival

Feeding regimes affected both length and dry weight from 30 dph, where larvae fed diet D2 were significantly different from the control group (*p =* 0.02), whereas larvae from group D1 showed no significant difference compared to the control or D2 group. From 50 dph, both dry weight (*p* = 0.01) and standard length (*p* = 0.03) became significantly different compared to the control group (*p* < 0.05). However, at 66 dph, the different protocols had no significant effect on the RGR and FCR. Survival was not affected by the different feeding regimes and averaged 21.3% (Table 4).

### 3.2. Skeletal Anomalies

**Figure 1:**
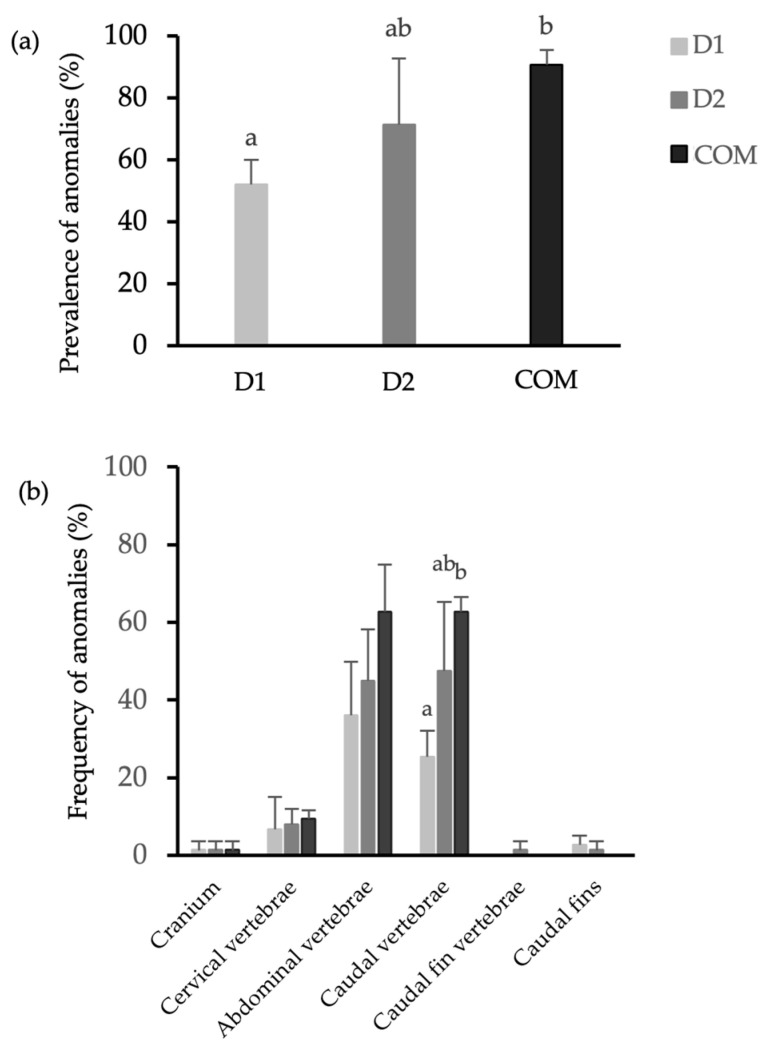
(**a**) Prevalence (%) of skeletal anomalies in Atlantic cod larvae; (**b**) frequency to region affected at 66 dph. Different lowercase letters indicate significant statistical differences (*p* < 0.05) between groups.

Atlantic cod larvae presented a relatively high prevalence of skeletal anomalies, ranging from 52 to 96% of larvae being affected by at least one skeletal anomaly. Among the dietary treatments, D1 demonstrated the lowest prevalence of anomalies (52%), significantly lower (*p* = 0.0444) than the prevalence observed in fish from the control group (91%) (Figure 1a). The frequency of skeletal anomalies across various anatomical regions in all experimental treatments is detailed in Figure 1b. Skeletal anomalies were mostly observed in the vertebral structures, with low prevalence in cervical vertebrae but a high prevalence in abdominal and caudal vertebrae. Notably, the prevalence of anomalies in the caudal vertebrae was significantly superior (*p* = 0.0335) in fish from the control group (63%) when compared to those in group D1 (25%). No anomalies were detected on dorsal or anal fins in any of the groups.

**Figure 2:**
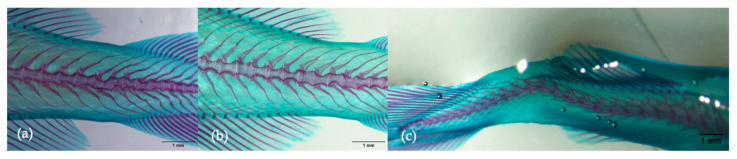
Examples of Atlantic cod larvae at 66 dph with (**a**,**b**) malformed vertebrae and (**c**) scoliosis. Scale bar is 1 mm.

Considering the count of anomalies per individual, larvae from group D1 exhibited a significantly greater (*p* = 0.015) proportion of individuals without skeletal anomalies (48%) compared to the control group (9.3%). Furthermore, severe anomaly occurrence was 48% lower in group D1 and 27% lower in group D2 when compared to the control group. The criteria for severe anomalies included scoliosis, lordosis, kyphosis, malformed jaws, three or more sequential vertebral fusions, deformed or reduced operculum, and the occurrence of more than three anomalies per individual. In the specific case of scoliosis (Figure 2c), larvae from the D1 group presented a 6.7% prevalence, significantly lower (*p* = 0.028) when compared to group D2 (36%) and the control group (64%) (*p* < 0.001).

### 3.3. Organ Ontogeny

The scoring described in Table 5 is composed of five organs and different indices adapted to each organ.

**Figure 3:**
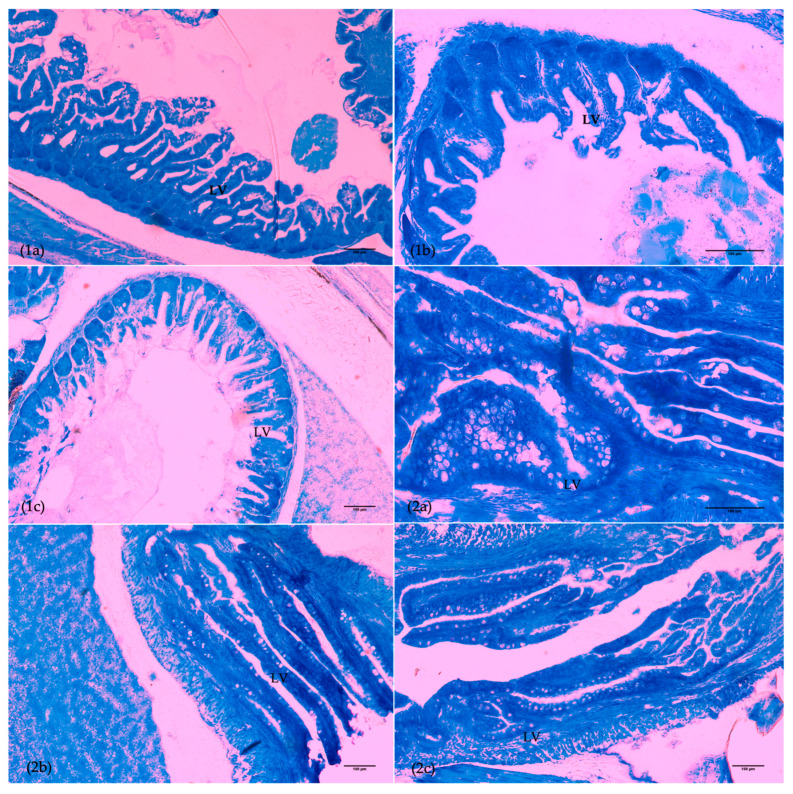
Transverse sections of anterior intestine (**1**) and posterior intestine (**2**) in a 66 dph Atlantic cod larvae from treatments D1 (**a**), D2 (**b**), and COM (**c**). LV, lipid vacuole. Scale bar is 100 µm.

Regarding the length of the villi on the anterior intestine (Figure 3), no significant differences were observed at 15 dph. However, at 30 dph, the length was significantly greater (*p* = 0.006) in D1 (119.5 µm) compared to D2 (107.8 µm). This trend continued at 66 dph, with significant differences between D1 (281.1 µm) and both the D2 (242.8 µm) (*p* < 0.001) and control (231.8 µm) groups (*p* < 0.001). Hence, this index score for group D1 was consistently superior to that of the other groups (Table 5). Goblet cells in the anterior intestine were only identified at 66 dph with a significantly higher abundance and consequent score in group D1 compared to the control (*p* = 0.006) and D2 groups (*p* = 0.004).

On the other hand, in the posterior intestine (Figure 3) at 15 dph, group D2 exhibited a lower ACLV than the remaining groups. However, this index was significantly higher in the D1 group compared to the control group at 30 dph (*p* = 0.04) and the D2 group at 66 dph (*p* = 0.019).

**Figure 4:**
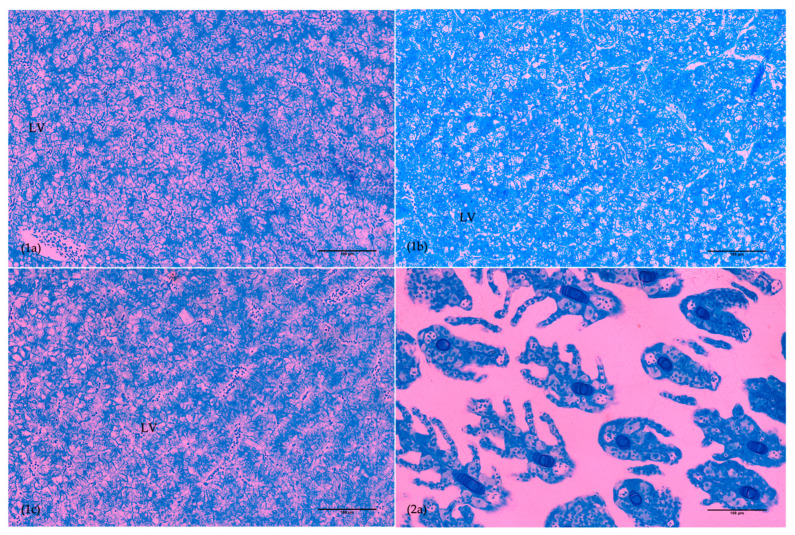
Transverse sections of anterior liver (**1**) at 66 dph and gills (**2**) at 30 dph in Atlantic cod larvae from groups D1 (**a**), D2 (**b**), and COM (**c**). LV, lipid vacuole. Scale bar is 100 µm.

The same index was evaluated in the liver (Figure 4) with a similar tendency at 15 dph as in the posterior intestine, and there was a significantly higher count (*p* < 0.001) in D1 compared to the other groups at 30 dph, reflected in a higher score.

Considering the ontogeny of the gills (Figure 4), according to observations at 15 dph, this organ was not apparent yet in the control group. However, the presence of erythrocytes was observed in both experimental groups. At 30 dph, group D1 had a significantly higher count of goblet cells compared to the control group (*p* = 0.045), whereas group D2 recorded a significantly higher count (*p* = 0.018) of hypertrophic chloride cells (HCC) than group D1. No edema or clubbing was observed in any of the groups. Moreover, fusion was lower in group D1 at all sampling points, especially compared to the results from group D2 (*p* = 0.003).

Regarding eye ontogeny, the score was higher in group D1 at 30 dph due to a tendency for better development of the pigment epithelium, photoreceptor layer, outer nuclear layer, outer plexiform layer, and inner nuclear layer. Additionally, the inner plexiform layer was significantly better developed (*p* = 0.019) in D1 compared to the D2 group at 30 dph.

## 4. Discussion

The effect of the feeding regime on growth, organ ontogeny, and skeleton anomalies of Atlantic cod was evaluated in this study. There was no positive effect of the experimental diets on growth, where the control group performed better after 30 dph, which could be due to the different physical properties of the microdiets. The microdiets D1 and D2 were observed to sink faster to the bottom of the rearing tank compared to the commercial control diet, which most likely affected availability. Standard length (SL) and dry weight (DW) were in the same range or higher as observed previously for protocols with the inclusion of natural zooplankton in the live feed phase [4,6,13,15,24,47,48]. For instance, Koedijk et al. [4] reported a SL of 2.5 cm and a DW of 20–25 mg at 60 dph, and in Folkvord et al. [6] zooplankton-fed larvae reached a DW of 30–35 mg at 65 dph. Also, in Vo et al. [48] cod larvae reached a SL of 2.4–2.7 cm and a DW of 20–30 mg at 65 dph. In our study, at 66 dph, cod attained 25–35 mg. These results are also higher than those of studies with experimental protocols including rotifers and *Artemia* spp. [1,17], which showed results of 2.2–2.5 cm SL/18–25 mg DW and 2.6–2.8 cm SL/25–30 mg DW, respectively, at 65 dph. Moreover, in studies with experimental microdiets with EFA being incorporated in the PL or NL fraction [28,29] and different PL sources [30], SL was in the range of 2.3–2.6 cm SL/15–23 mg DW at 65 dph. Moreover, the RGR was also comparable to previous studies with the inclusion of zooplankton [5,49] and microdiets with different PL sources [30]. Finally, the survival observed in this study was comparable to that reported in previous studies [4, 6 (average 26%), 15 (10–20%)] or higher [1 (4–8%), 28, 29 (12.2–15.7%), 17 (11–16%)]. Many types of zooplankton including copepods, barnacles, ciliates, polychaeta, and lamellibranchs are known to be part of the natural diet of early life stages of Atlantic cod [50,51,52], and the specific advantages of the use of copepods in cod larvae rearing have been emphasized and proven valuable for larval development, growth, and survival while providing the possibility of selecting different prey size [4,5,10,12,13,14,15,47]. In fact, prey size, distribution, and availability can limit the growth and development of Atlantic cod. However, besides ethology, the most determinant factor has proven to be the nutritional composition of the feed source used [13,48]. Therefore, the results obtained here with the inclusion of barnacle nauplii are in accordance with previous data from studies with natural zooplankton, which is supported by similarities in their nutritional profile [18,19,22]. Another factor that affects growth and development is the onset of the weaning phase and the establishment of a co-feeding period, which should be adapted to the development of the digestive system, assuring ingestion and digestion capacity [22,25,26,53,54]. Moreover, inert feeds should be attractive to the fish, taking into account physical and chemical factors while ensuring a nutritional balance [8,23,25]. For Atlantic cod larvae, some studies have tested experimental feeds with different sources and inclusion levels of PLs and NLs [17,24,29,30]. Microdiets with higher inclusion of PLs have been associated with better growth and survival of Atlantic cod larvae [29,48,54,55] and other species [34,56,57,58,59,60]. Furthermore, it has been discussed that cod larvae and other species better utilize fatty acids from the polar lipid fraction, having its dietary inclusion as a requirement [8,28,29,30,34,61]. Cod larvae have high requirements of EPA and DHA fatty acids, mostly in early development, which are more efficiently absorbed from dietary PLs [29]. Another advantage of PL addition is their ability to contribute to the cohesiveness of dietary microparticles, helping reduce the leaching of nutrients [62]. However, several studies have also demonstrated that not only quantity but also the origin of dietary PLs influences growth performance, survival, digestive enzyme production, plasma lipid profile, and lipoprotein metabolism [30,57,58,63,64]. In this study the different sources of PLs between experimental microdiets had no clear effect on growth or survival, with both groups being comparable to the control group. Results by Hansen et al. [62] suggested that higher PL diets can have a clearer positive impact on growth when introduced earlier and in co-feeding with *Artemia* spp. nauplii; however, the authors did not see benefits of feeding a higher PL diet alone. Nevertheless, growth performance results in the present study were higher than previously reported in cod larvae fed with diets supplemented with PLs, early weaning, and co-feeding with *Artemia* spp. [28,29,30,48,65].

In addition, according to Kjørsvik et al. [29], high-PL diets resulted in overall faster ossification of the vertebral column in cod larvae, and fin rays were in a more advanced stage of development at 45 dph in the group fed a higher PL diet when compared to a higher NL diet. In the same study, no significant differences in the occurrence of skeletal anomalies were observed. However, Cahu et al. [34] reported lower rates with the highest dietary PLs and suggested that the anomaly rate is related to the proportion of PLs:NLs in seabass (*Dicentrarchus labrax*). In the present study, group D1 exhibited a significantly lower prevalence of skeletal anomalies, including severe anomalies, which may suggest that its occurrence could also be related to dietary PL sources, as well as EFAs supplied as dietary PLs [8,66]. This is in line with results from Hansen et al. [30], where Atlantic cod fed with a diet richer in marine PLs had a higher abundance of swim bladder abnormalities at 35 dph and skeletal anomalies at 75 dph. Furthermore, the authors also reported that scoliosis occurrence was significantly lower in the vegetable PL group, in line with the present study. 

Besides skeletal development, histological analysis is considered a good tool to assess the dietary impact on fish development [28,62]. In addition to a lower prevalence of skeletal anomalies, fish from group D1 also showed an overall accelerated organ ontogeny when compared to group D2 and the control. The delayed development of the gills and anterior intestine at 15 dph in the control group could be attributed to the difference in the live feed protocol used until then, with the addition of Cryo-µ and Cryo-S. On the other hand, the delay in development of the posterior intestine, liver, and eyes in the control group, compared to D1 at later development stages, is likely due to beneficial effects of the D1 microdiet and its combination with the live feed protocol applied. Notably, the development index in the anterior intestine was also superior in the D1 group. When comparing experimental groups, D1 had an improved ontogeny compared to D2, at 30 and 65 dph, which could be attributable to the use of a higher proportion of vegetable PLs than marine phospholipids. It has been described that PL dietary levels positively influence digestive system maturation [34], as well as sources, since their effects vary with their components [8,28,30]. The results of Wold et al. [28] helped confirm the advantages of a high-PL diet for cod larvae growth and development and suggested that liver structure was the most sensitive to differences in lipid composition where hepatocyte size and nucleus size were significantly larger in the diets with higher PLs, which could be connected to higher metabolic activity and growth. On the other hand, that study recorded higher lipid accumulation in hepatocytes in fish fed with diets with higher NLs, and even though there were no significant differences in total lipid vacuolization in enterocytes, the authors suggested that dietary PL composition can affect it during earlier phases of cod larvae ontogeny. Hence, these authors suggest marine PLs are more beneficial, which is in apparent opposition to the tendency observed in the present study. However, not only the source but also the composition of fatty acids of each lipid class has effects on growth and development [8,28,29,30], and different sources have different compositions. So, the apparently contradicting results may be due both to the different proportions of marine and vegetable phospholipids used in the different studies, and to their composition in essential fatty acids. However, the present study only tested three microdiets with different fatty acid and phospholipid compositions. Clearly more studies are needed to refine the optimal nutritional composition of cod larval microdiets in terms of fatty acid and phospholipid, but also other nutrients such as amino acids, vitamins, and minerals. Moreover, it should be tested if an early introduction of microdiets, and/or longer co-feeding with cryoplankton, may bring performance and quality benefits to cod larvae.

## 5. Conclusions

In conclusion, our results highlight the potential of optimizing feeding protocols for Atlantic cod larvae and the positive effects this may have on larval and juvenile quality. Both novel live feed products and novel formulated diets bring clear benefits. In the present study, the D1 group, combining Cryo-µ, Cryo-S, Cryo-L, and rotifers as live feeds, with the subsequent introduction of microdiet D1, led to cod larvae with a lower prevalence of skeletal anomalies, including severe anomalies, and had a positive effect on organ ontogeny.

## Figures and Tables

**Table 1:** Feeding protocols timeline where D1 and D2 refer to the experimental groups and COM to the control group. dph—days post hatching.

dph	3		10		20		25	27		45		66
**COM**												
Enriched rotifers												
Cryo-L												
Commercial microfeed												
**D1 and D2 groups**												
Enriched rotifers												
Cryo-μ												
Cryo-S												
Cryo-L												
D1/D2 microfeed												

Note: Text in bold stand for the treatment groups; Different colours stand for different feed items

**Table 2:** Proximate composition, total phosphorus, DHA, EPA, and phospholipid (PL) levels of the 3 inert diets tested for weaning Atlantic cod larvae. D1 and D2 refer to the experimental groups and COM to the control group.

	D1	D2	COM
Protein (%DM)	63.0	62.5	58.3
Fat (%DM)	18.6	18.9	17.6
Ash (%DM)	10.4	10.1	13.4
Phosphorous (%DM)	1.4	1.4	1.6
EPA (%DM)	1.1	1.3	1
DHA (%DM)	1.4	1.3	1.3
Total PLs (%DM)	9.0	8.8	10.0
Marine PLs (% total PL) *	46	74	NA
Vegetable PLs (% total PL) *	54	26	NA

* Estimated based on analyzed composition of ingredients; NA—not available.

**Table 3:** List of considered skeletal anomalies in Atlantic cod larvae by region affected and type of anomaly.

Regions Affected
A. Cranium B. Cervical vertebra (vertebrae 1–2; short vertebra centra, prominent neural spines and absence of articulations with ribs) C. Abdominal vertebrae (vertebrae 3–19; vertebrae with wing-shaped transverse processes (parapophyses) that all articulate with a rib) D. Caudal vertebra (V20–V40; vertebra centra have haemal arches with prominent haemal spines E. Caudal fin vertebrae (V41 to the last vertebra; characterized by broad neural and haemal spines, providing sites of origin for muscles inserting on the fin rays—lepidotrichs—of the tail fin) F. Anal fin G. Caudal fin H. Dorsal fin
**Type of Anomaly**
* Scoliosis * Lordosis * Kyphosis ** Vertebral fusion Vertebral body malformation Malformed neural arch and/or spine Malformed haemal arch and/or spine and/or rib Malformed ray (deformed, absent, fused) Malformed pterygiophores (deformed, absent, fused) Malformed hypural (deformed, absent, fused) Malformed epural (deformed, absent, fused) * Jaw deformities Reduced dental/malformed pre-maxillary and/or maxillary Vertebral slipping * Deformed or reduced operculum

* Severe anomaly; ** three or more sequential criteria are considered a severe anomaly.

**Table 4:** Growth performance of Atlantic cod larvae from the control group (COM) and two experimental groups (D1 and D2) at 3, 30, 50, and 66 dph. Values are presented as mean ± standard deviation. Different superscript letters indicate statistical differences (*p* < 0.05) between groups at the same age.

dph	Group	Standard Length (cm)	Dry Weight (mg)	RGR (%/day) ^1^	FCR	Survival (%)
3	D1	0.45 ± 0.03	0.06 ± 0.01	-	-	-
	D2	0.45 ± 0.03	0.06 ± 0.01	-	-	-
	COM	0.44 ± 0.03	0.06 ± 0.01	-	-	-
30	D1	0.86 ± 0.05 ^ab^	0.54 ± 0.39 ^ab^	7.47 ± 0.99	-	-
	D2	0.85 ± 0.04 ^a^	0.47 ± 0.17 ^a^	7.57 ±1.20	-	-
	COM	0.88 ± 0.05 ^b^	0.54 ± 0.21 ^b^	6.78 ± 0.86	-	-
50	D1	1.38 ± 0.06 ^a^	3.42 ± 1.58 ^a^	8.36 ± 1.07	-	-
	D2	1.37 ± 0.05 ^a^	3.13 ± 1.61 ^a^	8.56 ± 0.99	-	-
	COM	1.57 ± 0.04 ^b^	4.79 ± 2.23 ^b^	8.64 ± 0.67	-	-
66	D1	2.62 ± 0.08 ^a^	25.6 ± 11.7 ^a^	9.61 ± 0.17	7.12 ± 0.44	21.4 ± 3.7
	D2	2.64 ± 0.08 ^ab^	27.3 ± 14.2 ^a^	10.0 ± 0.03	6.86 ± 0.80	17.7 ± 1.2
	COM	2.79 ± 0.07 ^b^	34.9 ± 16.4 ^b^	9.75 ± 0.08	5.40 ± 0.70	24.8 ± 0.6

^1^ Relative growth rate (RGR) values given refer to the interval between the initial and the present sampling points.

**Table 5:** Scoring system for the histological evaluation of Atlantic cod larvae in anterior intestine, posterior intestine, liver, eye, and gills, fed the three protocols. ACLV refers to an area covered with lipid vacuoles, HCC refers to hypertrophic chloride cells.

dph	Group	Anterior Intestine		Posterior Intestine	Liver	Eye	
		Villi Length	Goblet Cells	ACLV	ACLV	Ontogeny	
15	D1	3	-	4	2	3	
	D2	2	-	3	2	3	
	COM	2	-	4	2	3	
30	D1	4	-	4	4	4	
	D2	3	-	4	4	3	
	COM	4	-	2	2	3	
66	D1	5	5	4	4	3	
	D2	4	4	3	3	3	
	COM	4	4	4	4	3	
		** Gills **					
		** Goblet cells **	** Erythrocytes **	** Edema **	** Fusion **	** Clubbing **	** HCC **
15	D1	3	2	1	1	1	1
	D2	3	2	1	1	1	1
	COM	1	1	2	2	1	2
30	D1	3	2	1	1	1	1
	D2	2	2	1	2	1	1
	COM	3	2	1	2	1	2
66	D1	3	3	1	1	1	1
	D2	2	2	1	3	1	1
	COM	3	2	1	2	1	2

The histological evaluation was carried out using a Multiparametric Semi-Quantitative Scoring System (modified from Pacorig et al. [47]). Scores of 1–5 correspond to absent, scarce, moderate, abundant, or highly abundant, according to the analyzed structure.

## Data Availability

The data that support the findings of this study are available from the corresponding author upon reasonable request.

## Abbreviations

The following abbreviations are used in this manuscript:

dph	Days post hatching
EPA	Eicosapentaenoic acid
ARA	Arachidonic acid
Cryo-S	Small barnacle nauplii
EFAs	Essential fatty acids
DHA	Docosahexaenoic acid
PLs	Phospholipids
COM	Control group
D1	Experimental group 1
D2	Experimental group 2
Cryo-L	Large barnacle nauplii
Cryo-µ	Blue mussel eggs
SL	Standard length
DW	Dry weight
RGR	Relative growth rate
FCR	Feed conversion ratio
ACLV	Area covered by lipid vacuoles
MSSSs	Multiparametric Semi-Quantitative Scoring System
HCC	Hypertrophic chloride cells
